# Time evolution of radiation-induced EPR signals in different types of mobile phone screen glasses

**DOI:** 10.1007/s00411-019-00805-1

**Published:** 2019-07-01

**Authors:** Małgorzata Juniewicz, Bartłomiej Ciesielski, Agnieszka Marciniak, Anita Prawdzik-Dampc

**Affiliations:** 1 Department of Physics and Biophysics, Medical University of Gdańsk, Dębinki 1, 80-211 Gdańsk, Poland; 2 Department of Oncology and Radiotherapy, Medical University of Gdańsk, Dębinki 7, 80-952 Gdańsk, Poland

**Keywords:** EPR, Dosimetry, Glass, Retrospective, Mobile phone, Radiation

## Abstract

In this study, samples of smart phone touch screen glass sheets and tempered glass screen protectors were examined with respect to their potential application in the dosimetry of ionizing radiation. The glass samples were obtained from various phones with different types of glass. Electron paramagnetic resonance (EPR) spectra of the radiation-induced signals (RIS) are presented and their dose dependence within a dose range of 0–20 Gy. Despite the observed fading with time of the dosimetric components of the signal, the remaining RIS turned out to be strong enough for a reliable dosimetry even 18 month after irradiation. The study also shows that crushing of the glass sheets and water treatment of the samples have no effect on the background and dosimetric EPR signals.

## Introduction

The investigation of electron paramagnetic resonance (EPR) spectra of different materials exposed to ionizing radiation is a subject of research of many laboratories worldwide. The EPR technique can be a valuable tool for retrospective, non-destructive dosimetry in radiation accidents, in particular for mass casualty incidents resulting in the exposure of numerous people to ionizing radiation. In such situations, a quick and simple sampling method followed by a fast identification of the absorbed dose is needed for the triage of the victims and for planning an appropriate medical treatment of those exposed (Trompier et al. 2017). Human tissues, such as tooth enamel and bone, already proved to be useful in ex vivo EPR dosimetry (Trompier et al. 2009b; Fattibene and Callens 2010; Krefft et al. 2014; Kaminska et al. 2016; Kinoshita et al. 2018). However, their applicability is limited due to the obvious difficulty in sample acquisition. Initial studies on EPR dosimetry in nail clippings indicated potential large inaccuracies in reconstructed doses, due to the presence of confounding EPR signals generated mechanically in the samples by cutting, and due to the fading of the dosimetric signal caused by exposure of nails to water (Trompier et al. 2009a; Marciniak et al. 2018) or induction of obscuring EPR signals by light (Sholom et al. 2018; Marciniak et al. 2019). Therefore, artificial materials in the vicinity of exposed individuals as well as personal belongings could provide better dosimetric materials more convenient in usage, provided that they preserve any radiation-induced EPR signals. Various laboratory glassware samples (e.g., from Jena, Rasotherm, Thuring, window glass) exhibit specific EPR signals induced by irradiation, which differ depending on the chemical content of the investigated glass (Gancheva et al. 2006). Kortmis and Maltar Strmecki (2018) studied soda-lime glass samples from six different glass batches and demonstrated the influence of temperature on the fading of RIS components over time.

Bortolin et al. (2019) studied glass samples used for blood test tubes, to reveal illegal omission of radiation sterilization of the blood in glass by means of the thermoluminescence (TL) and EPR techniques. Both techniques allowed detection (up to 1 year after the exposure) of effects induced by high doses of ionizing radiation (10^3^ Gy) in the glass at the manufacture stage. Recently, commonly used electronic devices containing glass elements, such as watches, eyeglasses or mobile phones, were investigated by several researchers with regard to their potential suitability as EPR dosimeters (Teixeira et al. 2008; Trompier et al. 2009b, 2010, 2011a, b; Bassinet et al. 2010). Touch screen glass from mobile phones is a particularly attractive material taking into account its widespread use and non-invasive, easy, and fast sample preparation (Marrale et al. 2012). Trompier et al. (2009b) measured mobile phone screen glass sheets and observed changes in EPR line shape after irradiation, which proved generation of a radiation-induced signal (RIS) in this material. So far, most of the EPR research of irradiated glass from mobile phones has been carried out on Gorilla Glass^®^. A report of Fattibene et al. (2014) summarized the results of an international intercomparison dosimetry project, in which the parameters of calibration lines and detection limits for irradiated Gorilla Glass^®^ were compared between participating laboratories. Recently, Sholom and McKeever (2017) studied EPR spectra of protective glasses from various manufacturers of mobile phones. Modern smartphones contain different types of screens, which may differ with respect to the properties of their EPR spectra generated by radiation. The variability of the background signals (BGSs) in different glasses was presented in articles of Sholom and McKeever (2017) and Sholom et al. (2018). Moreover, it was shown by McKeever et al. (2018) that the BGS may differ between samples cut from different regions of the same screen. Sholom et al. (2018) presented two methods of dose reconstruction. Particularly, a higher intensity of EPR signals around the edge of the investigated screen was observed, which was assigned to an exposure to UV radiation during manufacturing.

A reliable retrospective dosimetry in real accidents requires also a knowledge regarding the time dependence of the dosimetric signals, as well as the effects of water and mechanical stress caused during preparation of the samples. Therefore, in the present study, results are presented regarding the time evolution of radiation-induced EPR signals by showing the kinetics of their decay during a long period—from a few hours up to 18 months after irradiation. The dose dependence of these signals in different types of glasses used in popular mobile phones is also presented, as well as the effects of water treatment and crushing of the samples on the EPR spectra. These are important practical factors in a potential post-accident dosimetry, when intact phones got irradiated and later their screens were exposed to water (e.g., in a rain or during cleaning) and crushed for EPR measurements.

## Materials and methods

The samples were obtained from four types of glass used for touch screens in mobile phones: Gorilla Glass (GG)—some of these samples had also been irradiated during a past intercomparison project (Fattibene et al. 2014) and other came from different batches: mineral glass (MG) from Sony Xperia L, model C2105, tempered glass (TG) used commonly as additional protective cover of the original screen—0.3 mm thickness, ninth level of hardness according to the Mohs’ scale, from Samsung S5, and screen glass obtained from iPhone 6S (IP).

**Fig. 1:**
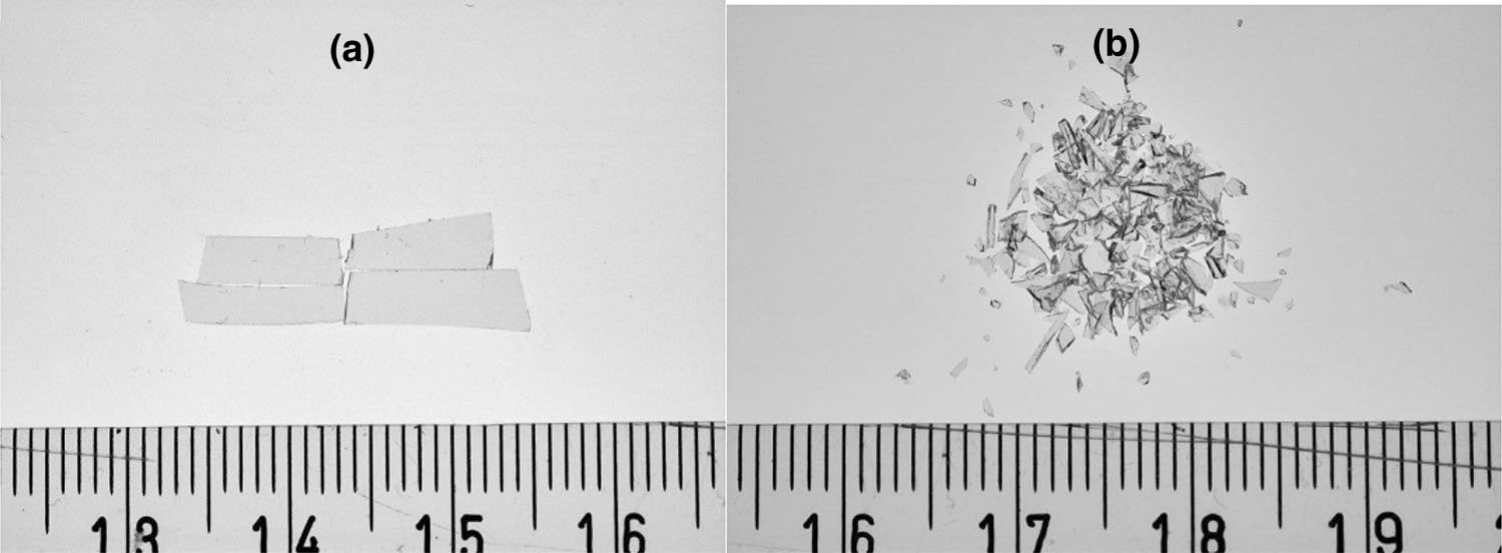
Microphotography of the mineral glass samples (MG_10 Gy) before (**a**) and after crushing (**b**). Their respective EPR spectra are presented in Fig. 2a

After separation of the glass parts from the LCD layers, the samples were washed with ethanol and crushed in a mortar into pieces of grain size of 0.3–4 mm. Some larger pieces were also measured to check the effect of crushing on the background signal (Fig. 1).

EPR measurements were performed with Bruker EMX 6/1 in X band with regular cavity type 4119HS W1/0430 at room temperature, using quartz sample tubes with 3 mm and 5 mm internal diameter. The following spectra acquisition parameters were applied: microwave power 32 mW, modulation amplitude 0.15 mT, sweep width 10 mT, conversion time 81.92 ms, and time constant 163.84 ms. As internal reference sample the marker ER 4119HS-2100 (Bruker BioSpin GmbH) was applied during all measurements, and the spectra were aligned and normalized with respect to the marker’s EPR line before further analysis. For every sample, 10 to 20 scans were averaged. The measurements were repeated three times at three different orientations of the sample in the cavity to minimize any potential effect of the samples’ anisotropy on the EPR spectra. The spectra were normalized to the mass of the samples which was in the 100–250 mg range. The spectrum of the empty tube was subtracted before further analysis of the spectra.

Irradiations of the samples were performed in the Department of Oncology and Radiotherapy, Medical University of Gdańsk (Poland) with 6 MVp photons from a Clinac 2300 medical accelerator. The crushed samples were irradiated with doses of 0.8, 2.0, 4.0, and 10.0 Gy (Gorilla Glass); 4.0, 8.0, 10.0, and 20.0 Gy (mineral glass); 2.0, 4.0, and 8.0 Gy (tempered glass); and 10.0 Gy (iPhone 6S).

The measurements of the background signals in the unirradiated samples were made at least 5 days after their crushing. The samples were stored at room temperature (about 24 °C) in the darkness; only the samples from the intercomparison project were kept in normal laboratory light conditions (but not exposed to direct sunlight) before and after irradiation.

For quantitative analysis of the spectral components, the same analytical method which was used by participants of the intercomparison project (Fattibene et al. 2014) was also applied in the present study. More specifically, the spectra of the irradiated samples were numerically separated into two benchmark model spectra: one was the background spectrum (i.e., that measured in the unirradiated sample) and the other was a model RIS spectrum obtained by subtraction of the background spectrum from the spectrum measured in the same sample irradiated with the highest dose (10 or 20 Gy). The same method was also used in Sholom et al. (2018) and McKeever et al. (2018). The magnitudes of the radiation-induced signals presented in the “Results and discussion” refer to the contributions of the model RIS components in the experimental spectra, calculated by numerical decomposition of the spectra. The decomposition was performed using the Reglinp procedure in the MS Excel package. The uncertainties (error bars) presented in the figures below refer to one standard deviation and reflect the repeatability of the EPR measurements (at three orientations of the sample tube in the cavity).

## Results and discussion

### Effect of crushing and water treatment

The marker line was removed from all spectra presented below, due to subtraction of the empty tube spectrum.

**Fig. 2:**
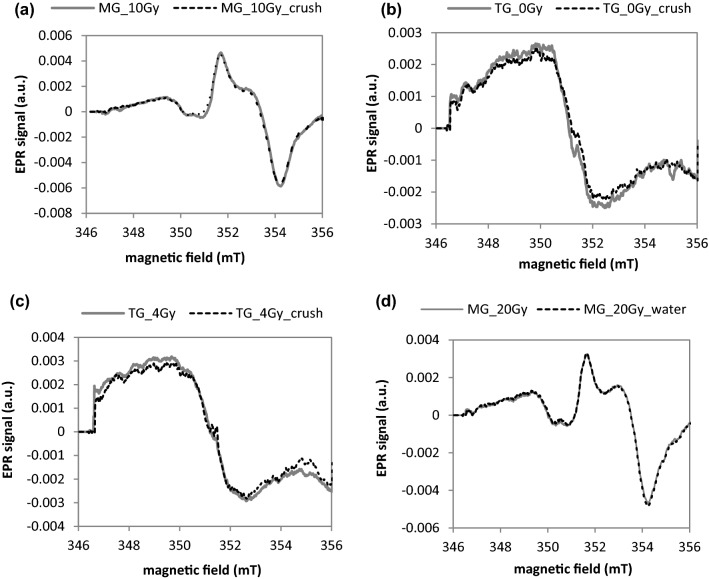
**a**–**c** Effect of crushing of unirradiated and irradiated samples on their EPR spectra; gray solid lines—large piece samples; black dotted lines—sub-millimeter grains. **d** Effect of water treatment on EPR spectrum of mineral glass phone screens irradiated to 20 Gy; solid line—before washing; dashed line—after 10 min of washing. *MG* mineral glass, *TG* tempered glass

Figure 2a–c shows the effect of crushing of the unirradiated and irradiated samples on the shapes of their EPR spectra. The spectra were first measured in large pieces (about 16 × 3 mm^2^, like those shown in Fig. 1a), and then measured again about 10 min after crushing the large pieces into sub-millimeter grains (like those shown in Fig. 1b).

Figure  2d shows the effect of immersion of the sample for 10 min into water for the glass sample irradiated to 20 Gy before the treatment.

The spectra presented in Fig. 2 prove that crushing of the glass samples to sub-millimeter grains did not affect their EPR signals—in both unirradiated (MG and TG) and irradiated (TG) samples, even when the samples were measured directly after crushing. This is in accordance with data of Bassinet et al. (2010), who reported a change in shape of the background signal and an increase in its intensity after grinding glass samples to a fine powder with grains below 315 μm, but did not observe any changes when grinding glass samples to larger grains. This mechanically induced increase in intensity of the background spectra in grains smaller than 315 μm decayed in about 10 h after crushing, as was shown by Trompier et al. (2011a). Furthermore, exposure of the irradiated MG sample to water did not induce any changes in its EPR spectrum. These results are important for practical applications of EPR dosimetry, because washing and crushing are necessary, indispensable steps in preparation of samples for EPR measurements. Also, in retrospective dosimetry an unintended exposure of the samples to water cannot be excluded. Therefore, the sensitivity of the EPR signals to water would be a potential, serious confounding factor.

## The effect of irradiation

**Fig. 3:**
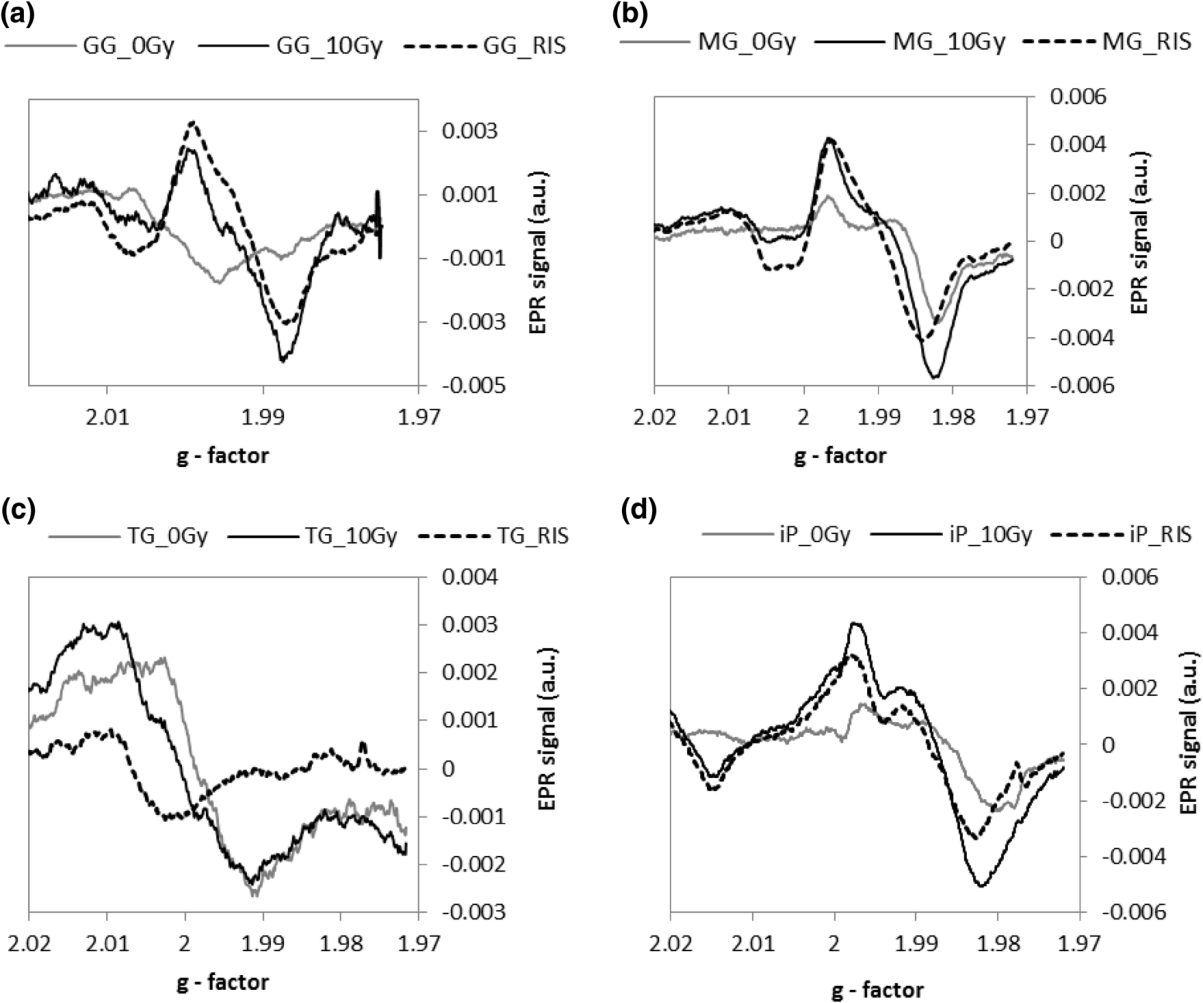
EPR spectra for unirradiated (gray lines) and irradiated (black solid lines) mobile phone glasses. The characteristic radiation-induced signals (RISs, dashed lines) were obtained by subtraction of the background spectra from the spectrum of irradiated sample. **a** *GG* Gorilla Glass, **b***MG* mineral glass, **c** *TG* tempered glass, **d** *iP* iPhone glass

Figure 3 shows the effect of a dose of 10 Gy on the EPR spectra for Gorilla Glass (A), mineral glass (B), tempered glass (C), and iPhone glass (D). The background spectra (0 Gy) of the four examined glass samples presented in Fig. 3 differ significantly. This variability imposes a serious limitation on the practical application of EPR dosimetry in screen glasses in real exposure scenarios when samples of unirradiated glass of the same type are not available. Moreover, as shown by McKeever et al. (2018), the intensity of the background signals from different locations of a screen may differ, which creates additional problems in accurate determination of the radiation-induced signal components.

**Fig. 4:**
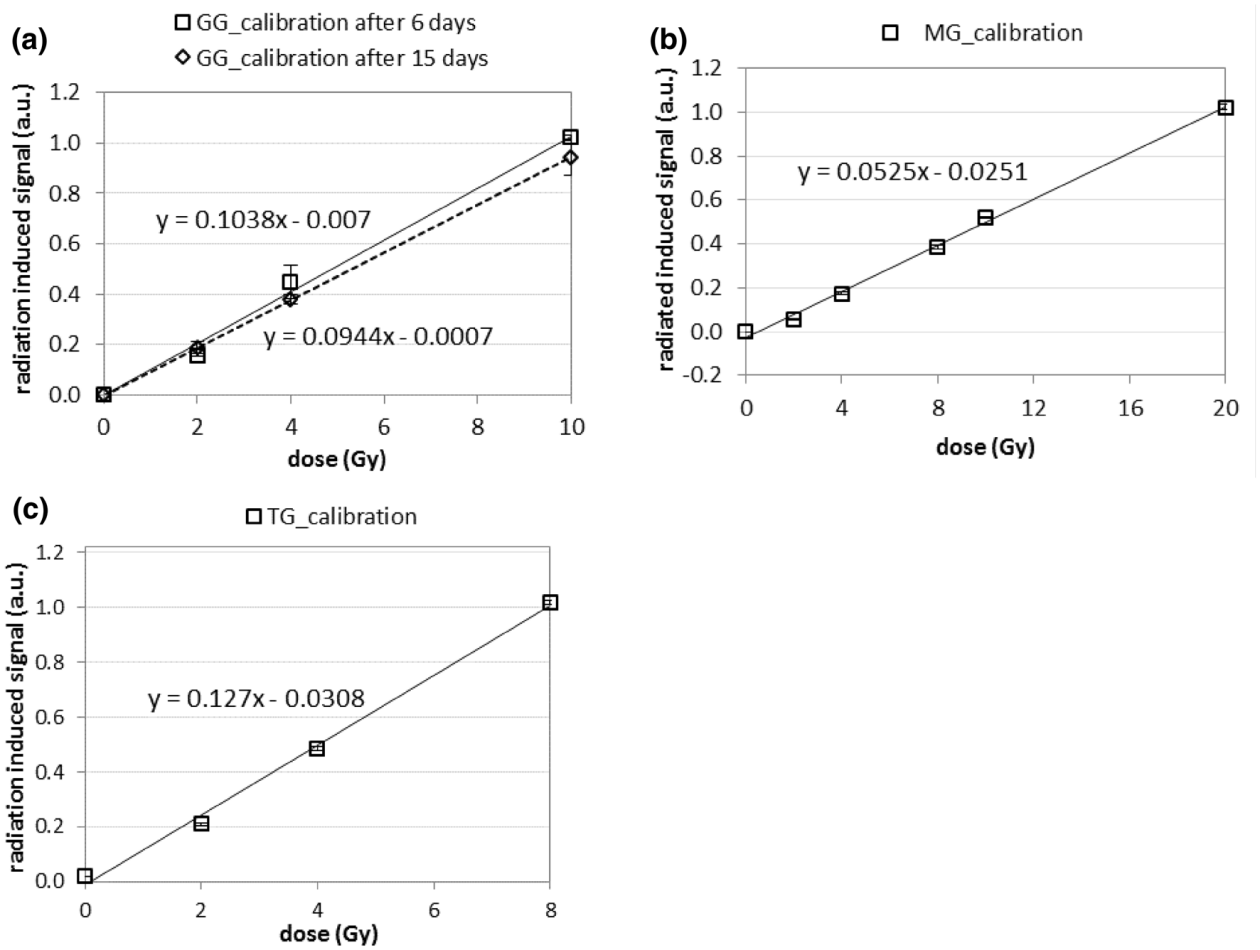
Dose dependence of radiation-induced signals for different types of the glasses. **a***GG* Gorilla Glass, **b***MG* mineral glass, **c***TG* tempered glass, solid and dashed lines—linear regressions of the data; error bars represent one standard deviation

The first EPR spectra for the irradiated samples were measured 6 days after irradiation for GG, 5 days for MG, 5 days for TG, and 10 days for iP. The data obtained suggest similar EPR spectra of paramagnetic centers produced by radiation in the MG, GG and, to a certain extent, IP samples—the shape of the spectra and the g factors of the dominating line (below 2.00) allow to identify the paramagnetic centers as the electron E centers reported by Sholom et al. (2018) and McKeever et al. (2018), accompanied by the presence of a lower intensity component at *g* > 2.00 which can be attributed to the presence of H centers. Also, the magnitude of the RISs is similar, suggesting that the radiation-induced defects may have a similar structure in these materials. In contrast, the RIS spectrum in the TG sample is completely different to that of the other samples, showing only one broad line at a *g* factor above 2.00, suggesting assignment of this paramagnetic center to one of the hole centers (probably the H2 center), without any spectral lines at *g* < 2.00. The amplitude of the RIS induced in the TG sample by 10 Gy is also significantly smaller in comparison to that of the other three glasses. Nevertheless, on the basis of the calibration line shown in Fig. 4, the precision of dose determination in the TG sample would be similar to that in the other materials—the dependence of the RIS on dose is monotonic with similar uncertainties in parameters of the regression lines (i.e., uncertainties in their slopes and intercepts).

**Fig. 5:**
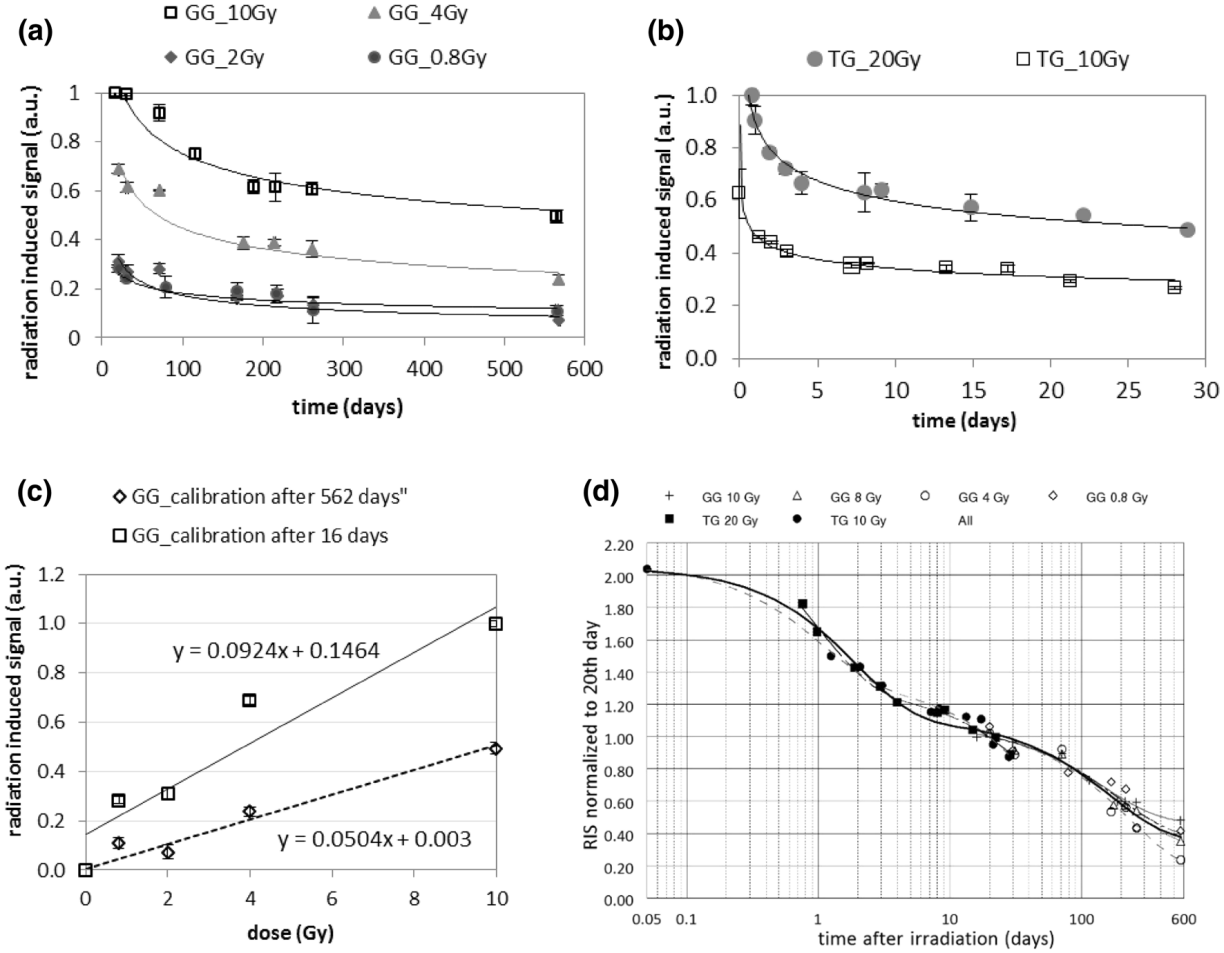
Changes in magnitude of the dosimetric signal with time for **a** four Gorilla Glass (GG) samples (that were also used by Fattibene et al. 2014); **b** two tempered glass (TG) samples; **c** calibration lines for GG are for data measured on 16th and 562nd day after irradiation; **d** time evolution of the dosimetric signals for TG samples (filled symbols) and GG samples (open symbols) normalized at the 20th day after irradiation; error bars represent one standard deviation

Figure 4 shows the dose dependence of the RISs for Gorilla Glass samples measured 6 days and 15 days after irradiation (Fig. 4a), mineral glass (Fig. 4b) and tempered glass (Fig. 4c) samples measured 5 days after irradiation. The solid lines represent a linear regression of the data. For all samples, within the studied dose ranges, the dose dependence is linear. The dose response curves of Gorilla Glass (Fig. 4a) measured 6 days and 15 days after irradiation slightly differ. The slope of the regression lines for samples measured after 15 days is lower than that for samples measured 6 days after irradiation. This may suggest the decay of the RIS in the time period between these two measurements. Such decay of the RIS in the first 2 weeks after irradiation was demonstrated for the TG samples (Fig. 5b) showing a rapid decay in RIS within the first 10 days after irradiation.

Figure 5 presents variations of the dosimetric signal (the RIS) with time after irradiation for two types of glasses (Gorilla Glass and the tempered glass) irradiated to various doses. The long-term decay of RIS in the GG samples can be roughly approximated by single exponential decay.

$$a0 + a1 \cdot {\text{e}}^{ - x/a2}$$

with the decay time *a*2 in the range of 150–230 days (*R*^2^: − 0.98). The fading of RIS in TG can be approximated with a high correlation coefficients *R*^2^ = 0.99 by two exponential decay.

$$a0 + a1 \cdot {\text{e}}^{ - x/a2} + a3 \cdot {\text{e}}^{ - x/a4}$$

with the slow decay constant *a*2 of about 70 days and the fast decay constant *a*4 of about 1 day. The fitted parameters are presented in Table 1.

**Table 1:** Fitted parameters for data in Fig. 5a, b

Fitted parameter	GG 10 Gy	GG 4 Gy	GG 2 Gy	GG 0.8 Gy	TG 20 Gy	TG 10 Gy
a0	0.479	0.210	0.046	0.099	0.034	0.041
a1	0.63	0.51	0.28	0.19	0.67	0.37
a2 (days)	146	197	235	188	76	61
a3	–	–	–	–	0.64	0.23
a4 (days)	–	–	–	–	0.97	1.0
*R* ^2^	0.982	0.977	0.960	0.902	0.994	0.988

*GG* Gorilla Glass, *TG* tempered glass

Figure 5a shows the change in the EPR signals with time for the irradiated GG samples, however, for a longer time scale (from 16 to 560 days after irradiation) than that for the TG samples. Exponential fitting of the data in Fig. 5a, b did not reveal any statistically significant differences in the decay kinetics between the GG and TG samples irradiated with different doses. Due to the difference in time scale for GG and TG (Fig. 5a, b), however, any reliable quantitative comparison of the decay kinetics between GG and TG is not possible. If one assumes that the signal fading observed in the GG and TG samples follows the same kinetics and if one normalizes the data for the GG and TG samples at the 20th day after irradiation (i.e., at a time point included in both sets of data), then the fading of the RIS can be illustrated as shown in Fig. 5d, in which the black solid line represents the fit of the data points for the all samples using the two exponential function mentioned above, with the slow (*a*2) and fast (*a*4) decay constants of about 170 and 1.9 days, respectively. Taking into account the uncertainty of the fit, these values are in rough agreement with the decay constants calculated separately for the GG and TG samples (i.e., about 150–230 days (GG) or 70 days (TG) for the slow decay, and about 1 day for the fast decay (in TG); see Table 1). However, more experimental data are necessary to validate the possibility of approximating the RIS decay in different glasses and for different doses by one joint mathematical function like the one proposed in Fig. 5d. The decrease in RIS amplitudes with time observed in all types of glass examined in the present study clearly demonstrates the need, for retrospective EPR dosimetry, to take into account the kinetics of signal fading. During the first 10 days after irradiation, the absolute loss in the RIS is about the same as during the following year. The decay curve in Fig. 5d allows to estimate roughly that during the whole observation period of almost 19 months the initial RIS (measured about 1 h after irradiation) decreased to about 20% of its initial value.

The effect of the resulting decrease in the slope of the dose calibration with time is illustrated in Fig. 5c, which shows the difference between the calibration lines for the GG samples measured 16 and 562 days after irradiation. Despite the significant decrease in the RIS in the GG samples during this period, the dosimetric EPR signal is still clearly seen, shows an evident, monotonic increase with dose, and thus can be useful for dosimetry in a dose range of several Gy, even delayed in time by many months after irradiation. The fast initial decay of the RIS is a strong contraindication for dosimetry in samples irradiated less than 5 days before measurement. For samples measured later than 5–10 days after irradiation, the signal calibration curve should be corrected for long-term decay, for example, using an approximation like the one given in Fig. 5d.

## Conclusion

In the present study, glass samples from different types of mobile phone screens were investigated. The results obtained show that glass screens from mobile phones can provide a good detector material in accident dosimetry. The EPR signals (background and the radiation-induced) are resistant to water and mechanical stress (crushing, cutting). Despite the observed decay of the RIS, dosimetry using mobile phone glasses is possible even 18 months after irradiation, due to the decay process which becomes much slower after about 5–10 days as compared to the initial fast decay. However, a serious limitation in accuracy of glass used for retrospective EPR dosimetry arises due to the variability in background signals between different glass samples. Solving this problem requires additional research focused on (1) the nature of this variability and (2) methods to separate the radiation-induced spectral EPR components from background, in EPR spectra of irradiated samples.
